# SUGAN: A Stable U-Net Based Generative Adversarial Network

**DOI:** 10.3390/s23177338

**Published:** 2023-08-23

**Authors:** Shijie Cheng, Lingfeng Wang, Min Zhang, Cheng Zeng, Yan Meng

**Affiliations:** 1 School of Artificial Intelligence, Hubei University, Wuhan 430062, China; 2 School of Computer Science and Information Engineering, Hubei University, Wuhan 430062, China; 3 Key Laboratory of Intelligent Sensing System and Security (Hubei University), Ministry of Education, Wuhan 430062, China; 4 Department of Land Surveying and Geo-Informatics, The Hong Kong Polytechnic University, Hong Kong, China

**Keywords:** generative adversarial network, image generation, mode collapse, training stability, gradient normalization

## Abstract

As one of the representative models in the field of image generation, generative adversarial networks (GANs) face a significant challenge: how to make the best trade-off between the quality of generated images and training stability. The U-Net based GAN (U-Net GAN), a recently developed approach, can generate high-quality synthetic images by using a U-Net architecture for the discriminator. However, this model may suffer from severe mode collapse. In this study, a stable U-Net GAN (SUGAN) is proposed to mainly solve this problem. First, a gradient normalization module is introduced to the discriminator of U-Net GAN. This module effectively reduces gradient magnitudes, thereby greatly alleviating the problems of gradient instability and overfitting. As a result, the training stability of the GAN model is improved. Additionally, in order to solve the problem of blurred edges of the generated images, a modified residual network is used in the generator. This modification enhances its ability to capture image details, leading to higher-definition generated images. Extensive experiments conducted on several datasets show that the proposed SUGAN significantly improves over the Inception Score (IS) and Fréchet Inception Distance (FID) metrics compared with several state-of-the-art and classic GANs. The training process of our SUGAN is stable, and the quality and diversity of the generated samples are higher. This clearly demonstrates the effectiveness of our approach for image generation tasks. The source code and trained model of our SUGAN have been publicly released.

## 1. Introduction

In recent years, there has been an ongoing intense competition between diffusion models [1,2,3] and generative adversarial networks (GANs) [4] in various domains, including image generation [5,6,7,8], image super-resolution [9,10,11,12], style transfer [13,14,15,16], image transformation [17,18,19], image-to-image translation [20,21,22], and adversarial attack [23,24]. This competition has greatly pushed and is continuously pushing the development of generative models. The ultimate goal of both types of models is to minimize the gap between the generated distribution and the target distribution. Diffusion models approach this by iteratively adding noise and denoising, while GANs employ adversarial training between the generator and the discriminator. Although diffusion models have been widely used in data-intensive tasks because of their stable sampling and training processes, their complex computations result in long training time and large space overhead. Moreover, due to their relatively fixed model structure, compared to GANs, diffusion models have limitations in flexibly handling different data types and task scenarios [25]. The above two are the main reasons why GANs are still being actively studied [26,27,28,29,30,31,32,33].

At present, how to improve the training stability and generation ability of GANs at the same time is one of the main challenges faced by GANs [34]. Adjusting the total parameters of the models in various scenarios is not a viable solution. Increasing the number of parameters can enhance the model’s feature extraction capability and potentially improve the quality of generated images. The representative models in this practice include DCGAN [35], LSGAN [36] and BigGAN [37]. However, excessive parameters may result in unstable training. This is why training instability is one of the biggest problems faced by these GAN models. Reducing the number of parameters can lower training difficulty. Many lightweight GAN models, for example, TinyGAN [38] and PGGAN [39], follow this practice. However, this cannot guarantee the extraction of important feature information from images, i.e., this can increase training stability, but can also result in decrease in the quality of generated images. Changing training strategies may be an alternative solution. For example, SAGAN [40] and StyleGAN [41] use the two time-scale update rule (TTUR) to update generators and discriminators by carefully adjusting the stride and frequency of updates for the learning rate. However, these methods greatly increase the workload of the hyperparameter optimization task and the difficulty of training, which may eventually result in unstable training [25,34]. Inspired by recent studies [5,7,14,22,27,42], we believe that by introducing a suitable normalization strategy, it is possible to effectively balance the training stability and image generation quality of GANs. Therefore, in this paper, we try to realize such a GAN model by enhancing the training stability of an existing powerful GAN model (which is with strong image generation ability) through the introduction of a suitable normalization method, while do not compromise the quality of its generated images.

U-Net [43] has been extensively studied since its publication in 2015. Its integration with GANs has greatly promoted the development of style transfer and image-to-image translation. Representatives of such models include Pix2Pix [44] and CycleGAN [45]. However, it has not been used in GANs for image generation tasks until recent years. Prior to the development of U-Net GAN [46], it was difficult to generate realistic images with fine details using GANs for image generation tasks. This is because GAN models usually rely on convolution operations for feature extraction. If the convolution kernel is too large, local features of the input image may be overlooked, resulting in blurred details. Conversely, if the convolution kernel is too small, global features of the input image may be disregarded, resulting in the generation of unrealistic images. In most cases, the global realism of the generated images is given higher priority over local details, so previous GANs tend to choose a large kernel size to ensure the global realism. By introducing the U-Net architecture [43] into its discriminator, U-Net GAN [46] can simultaneously focus on both global and local information in images. This capability allows U-Net GAN to simultaneously learn the differences in both global and local features, which enables it to generate images that are realistic both globally and locally. The U-Net architecture also shows a positive impact on controlling the number of network parameters. Because of the above merits, the images generated by U-Net GAN have a more varying structure, appearance, and delicate details than the famous BigGAN [37]. However, the spectral normalization [47] used in the discriminator of U-Net GAN is difficult to function when dealing with a large number of training parameters and complex datasets, which results in a serious mode collapse problem.

In this paper, we propose a stable version of U-Net GAN (SUGAN) by introducing gradient normalization [48] to the state-of-the-art GAN model U-Net GAN, improving its training stability while keeping its high image generation capability. The proposed SUGAN was compared with several state-of-the-art and classic GANs using multiple image datasets for both unconditional and conditional image generation tasks. The results show that our SUGAN outperforms other models in terms of the evaluation metrics of Inception Score [49] and Fréchet Inception Distance [50]. Additionally, the generated images by our SUGAN exhibit a higher level of realism, while the training process is stable. The source code and trained model of our SUGAN have been publicly released at https://github.com/ChengsjCV/SUGAN (accessed on 17 August 2023).

## 2. Related Work

### 2.1. U-Net GAN

U-Net GAN [46] uses U-Net [43] as the main body of the network architecture for the discriminator. The U-Net-based discriminator consists of an encoder for feature extraction and a decoder for detailed per-pixel analysis of the images, prompting the generator to focus on both the global and local consistency between generated and real images. At the same time, skip connections are used to realize feature fusion to retain more texture and spatial information. The network is also simple in structure and small in number of parameters. With the help of this structure, the performance of the discriminator is greatly improved, making the generator’s task of cheating the discriminator more difficult, thus improving the performance of the generator.

Although U-Net GAN improves the quality of generated images by modifying the discriminator architecture to learn the global and local pixel differences between real and generated images, it suffers from serious mode collapse when dealing with complex datasets. This issue arises due to the utilization of Jensen–Shannon divergence (JS-divergence, see Formulas (1) and (2)) as measure to evaluate the distance between real and generated distributions.

(1) $$KL(P_{1}||P_{2})=E_{x\sim P_{1}}\log\frac{P_{1}}{P_{2}}$$

(2) $$JS(P_{1}||P_{2})=\frac{1}{2}KL(P_{1}||\frac{P_{1}+P_{2}}{2})+\frac{1}{2}KL(P_{2}||\frac{P_{1}+P_{2}}{2})$$

where *P*_1_ and *P*_2_ represent two data distributions, and *x*~*P*_1_ is the sample that follows *P*_1_ distribution.

The objective function of U-Net GAN is as follows:

(3) $$\underset{G}{\min}\underset{D}{\max}V(D,G)=E_{x\sim p_{data}(x)}[\log D(x)]+E_{z\sim p_{z}(z)}[\log(1-D(G(z)))]$$

where *x* is a real sample, *z* is a random noise, and *D*(*x*) represents the output of both the encoder and the decoder of U-Net GAN. Goodfellow et al. [4] proposed that when the generator is fixed, the optimal discriminator is:

(4) $$D_{best}(x)=\frac{P_{data}(x)}{P_{data}(x)+P_{g}(x)}$$

where *P_data_* and *P_g_* represent the distributions of real data and generated data, respectively. In this case, the optimization goal of the generator is:

(5) $$\underset{D}{\max}V(D,G)=E_{x\sim p_{data}(x)}[\log D_{best}(x)]+E_{z\sim p_{z}(z)}[\log(1-D_{best}(G(z)))]$$

When there is no overlap between the real data distribution and the generated data distribution, the result of Formula (5) will be a constant, and then the generator cannot be trained because of the vanishing gradient problem, resulting in a training failure [49,51]. To fix the problem of unstable training for U-Net GAN, in this paper, we introduce gradient normalization to its discriminator, and obtain a stable version of it (we name it stable U-Net GAN).

### 2.2. Normalization in GANs

Studies have shown that adding normalization to the discriminator can have a good effect on improving the training stability of GANs [47,52,53,54]. These approaches usually ensure training stability by controlling weights or gradients.

In terms of controlling weights, common practices include batch normalization [52], L2 normalization [53] and weight clipping [54]. They improve the generative performance of the model by constraining the values of weights. However, these constraints also limit the model capacity of the discriminator, making it unable to fully learn the features of the input data, and thus unable to accurately identify real and generated images. Therefore, these strategies are often used in GANs for simple and low-resolution datasets.

In terms of controlling gradients, the existing normalization methods often formulates the objective function of the discriminator as a continuous function bounded by a fixed Lipschitz constant K [55,56]. This ensures that the gradient space of the discriminator is continuous and smooth, and thus improve the training stability of GANs. Wu et al. [48] classifies Lipschitz constraints into three categories:

1. Model-wise or module-wise constraints. Model-wise constraints depend on full model while the module-wise constraints depend on the sum of internal modules.
2. Sampling-based or non-sampling-based constraints. If a constraint approach requires sampling from a fixed pool of data, it is called a sampling-based constraint, otherwise it is called a non-sampling-based constraint.
3. Hard or soft constraints. When constraining the gradient norms of any function in the discriminator, if none of these values is greater than a fixed value, the constraint is called a hard constraint, otherwise it is called a soft constraint.

Among the three categories of constraints mentioned above, module-wise constraints limit the performance of the current layer, sampling-based constraints are ineffective for the data that are not sampled before, and soft constraints cannot guarantee gradient stability during training because of the inconsistency of gradient norms. To sum up, in terms of controlling gradients, a model-wise, non-sampling-based and hard constraint is the optimal choice. Common normalization methods that control gradients include gradient penalty [57,58,59] and spectral normalization [47,60,61]. Gradient penalty is a model-wise, sampling-based, and soft constraint, while spectral normalization is a module-wise, non-sampling-based, and hard constraint, neither of which is the optimal choice for Lipschitz constraints. Besides, they also suffer from hyperparameter sensitivity and require additional tuning work on different datasets, resulting in poor generalization ability.

The gradient normalization proposed by Wu et al. [48] is a model-wise, non-sampling-based, and hard constraint. It not only satisfies the Lipschitz constraints optimally but also reduces the difficulty of the tuning tasks by avoiding the introduction of additional hyperparameters, further stabilizing the training process of the model. Besides, it is suitable for different GAN architectures, which enables the model to process datasets of different scenarios and resolutions, thus further improving the stability and generation ability of the model. All these above are the reasons why we introduce gradient normalization instead of other normalization methods to the U-Net GAN for better training stability. Table 1 shows the comparison of gradient normalization (GN), gradient penalty (GP), and spectral normalization (SN) in terms of the Lipschitz constraints.

## 3. Methods

The state-of-the-art model U-Net GAN [46] can generate fine-grained images with clear textures. However, it may suffer from serious mode collapse. Gradient normalization can enhance the training stability of GANs and their ability to fit datasets, effectively alleviating the mode collapse problem. Therefore, we develop a stable U-Net GAN (SUGAN) by introducing gradient normalization to the discriminator of the original U-Net GAN. Compared with the original model, the proposed SUGAN exhibits superior generative performance, enhanced training stability and more diverse generated sample styles, i.e., the mode collapse problem of the original U-Net GAN is well alleviated while the high image generation capability is well kept.

The details of the generator, the discriminator, and the loss function of our SUGAN are presented in Section 3.1, Section 3.2, and Section 3.3, respectively.

### 3.1. Generator Architecture

**Figure 1:**
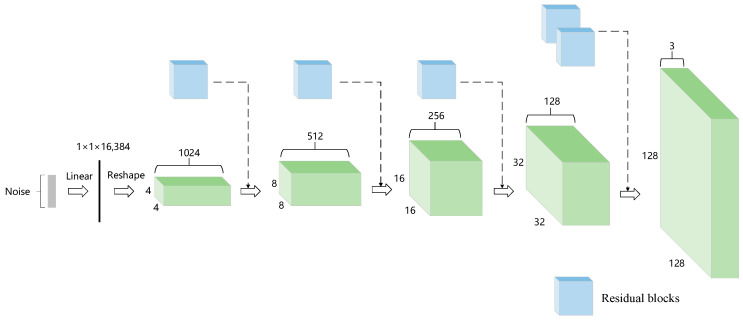
The overall structure of the generator of our SUGAN. The input to the model is a noise, which represents a random latent vector. The blue blocks represent the residual blocks, and the green blocks are used to demonstrate the shape change process of the input noise vector.

**Figure 2:**
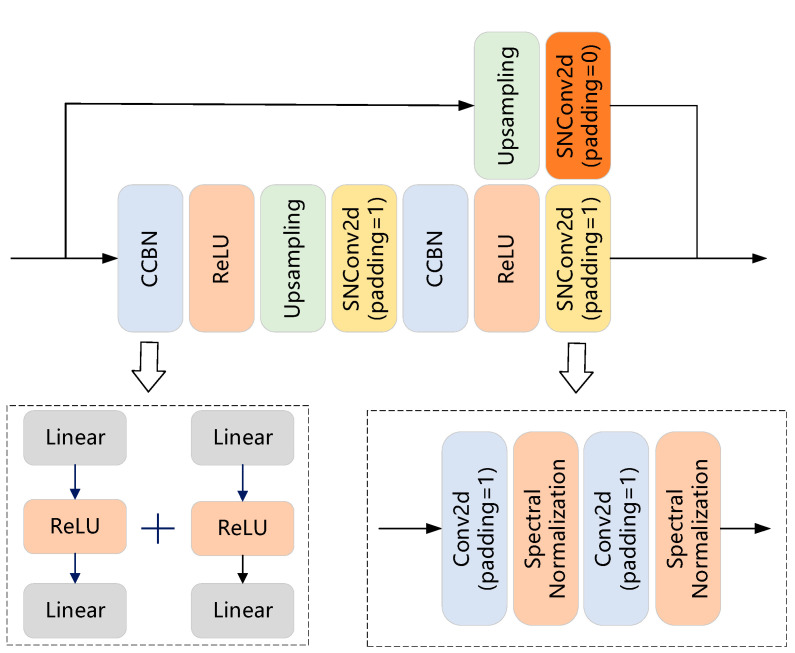
The structure of the residual block in the generator of our SUGAN. This residual block consists mainly of four types of layers: CCBN, upsampling, SNConv2d, and ReLU. The difference between the two kinds of SNConv2d blocks is that their convolution kernel sizes are different. The convolution kernel size is 3 when the padding is equal to 1, and 1 when the padding is equal to 0.

Figure 1 shows the overall structure for the generator of the proposed SUGAN and the step-by-step transformation of the random noise into the generated image in the generator. The generator is mainly composed of residual blocks. The detailed structure of each residual block is shown in Figure 2, where each SNConv2d is a convolutional layer with spectral normalization and each CCBN represents a class-conditional normalization layer. Each CCBN layer consists of two identical fully connected blocks that adjust the number of channels in both unconditional and conditional image generation tasks, enabling the model to complete both generation tasks without changing the architecture. The upsampling layers in Figure 2 use nearest neighbor interpolation instead of transposed convolution for progressive upsampling.

**Figure 3:**
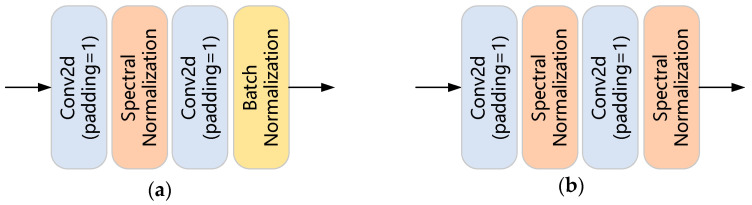
The structures of the SNConv2d layer in the generator of (**a**) the original U-Net GAN and (**b**) our SUGAN.

The SNConv2d layer in the generator of the original U-Net GAN consists of two different normalization layers (see Figure 3a): a spectral normalization layer and a batch normalization layer. Compared with batch normalization, spectral normalization performs better in controlling gradient explosion and reducing computational cost. Therefore, we modify the residual block in the generator of the original U-Net GAN by replacing all the batch normalization layers in SNConv2d with spectral normalization layers, so as to achieve the purpose of stable generator training.

### 3.2. Discriminator Architecture

**Figure 4:**
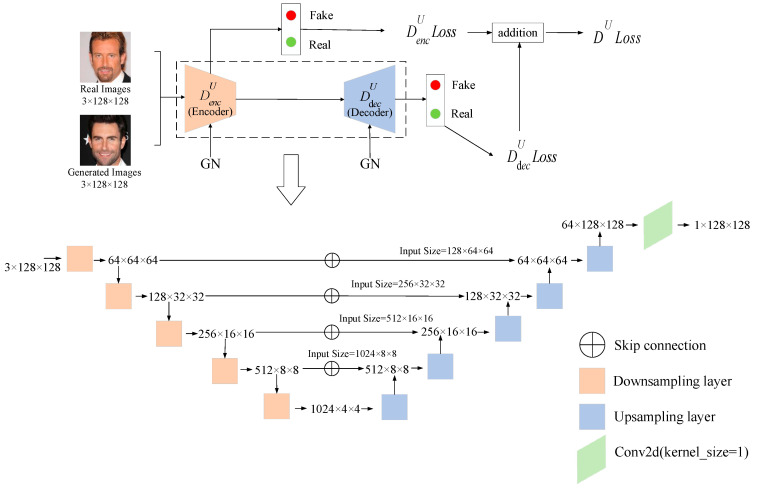
The overall structure of the discriminator of our SUGAN. Benefiting from the U-Net architecture, the discriminator can combine global and local features to ensure quality of generated images. In addition, the gradient normalization (GN) is used to ensure the training stability.

**Figure 5:**
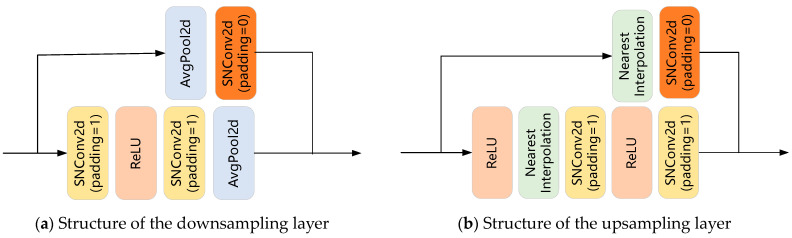
The structures of (**a**) each downsampling layer and (**b**) each upsampling layer in the discriminator.

The discriminator (see Figure 4 for overall structure) adopts the U-Net structure, consisting of an encoder and a decoder. The encoder mainly consists of downsampling layers (see Figure 5a) and the decoder mainly consists of upsampling layers (see Figure 5b). The operations corresponding to downsampling and upsampling are average pooling (see AvgPool2d in Figure 5a) and nearest interpolation (see Nearest Interpolation in Figure 5b), respectively. With the help of skip connection between the encoder and decoder (see skip connections in Figure 4), the extracted shallow features and deep features are fused, so that the discriminator can pay attention to both the global and local information at the same time, and thus stimulate the discriminator to learn their differences. When the performance of the discriminator is improved, the generator needs to generate samples of higher quality to fool the discriminator, thus improving the performance of the generator.

In this paper, we introduce gradient normalization to the training process of the discriminator to improve the training stability for the original U-Net GAN. As a model-wise, non-sampling-based and hard constraint (refer to [48] or see the brief review in Section 2.2), gradient normalization ensures that the gradient scales are the same and avoids the problem that some parameter updates may be too large or too small, which, otherwise, will result in the explosion or vanishing of gradients during the training process. This means the training stability of the proposed model will be improved compared with the original U-Net GAN in theory. Additionally, gradient normalization does not depend on the specific distribution of the data, making it applicable to various types of datasets. It should be noted that gradient normalization acts on the identification stage of real or fake images in the discriminator, while the generator does not have this stage, which is why batch normalization is not replaced by gradient normalization but by spectral normalization in the generator (see Section 3.1).

The formula for gradient normalization varies depending on the tasks. For the unconditional image generation task, it is calculated as shown in Formula (6).

(6) $$\overset{\wedge }{D}(x)=\frac{D(x)}{∥\nabla_{x}D\left(x\right)∥+\left|D(x)\right|}$$

where *D* and ∇*D*(*x*) represent the discriminator and the gradient of the input sample *x*, respectively.

For the conditional image generation task, it is necessary to introduce additional conditional information to the GAN model to assist the generation, and such information are usually class labels. In this task, the gradient normalization is calculated as shown in Formula (7).

(7) $$\overset{\wedge }{D_{y}}(x)=\frac{D_{y}(x)}{∥\nabla_{x}D_{y}\left(x\right)∥+\left|D_{y}(x)\right|}$$

where *D_y_* represents the discriminator with additional conditional information *y*. 

Compared with gradient penalty and spectral normalization, no extra hyperparameters are introduced into the calculation of gradient normalization. Therefore, among the three normalization methods (see Section 2.2), gradient normalization can minimize the issue of parameter sensitivity, and thus the difficulty of hyperparameter tuning is relatively minor for our SUGAN.

### 3.3. Loss Function

As stated above, similar to the original U-Net GAN, our SUGAN is composed of two subnetworks: a generator G and a U-Net-based discriminator $D^{U}$. Therefore, the discriminator of our SUGAN also includes two loss calculations: the loss calculation in the encoder is used to determine whether the input picture is real or fake, while the loss calculation in the decoder is to further provide a per-pixel feedback about real or fake to the generator. However, different from the original U-Net GAN, we introduce gradient normalization to the discriminator. It acts on the real or fake identification stage of the encoder and the decoder, helping the model stabilize the gradient and avoid the problems of gradient vanishing and gradient explosion.

Similar to other GANs, the core of our SUGAN is also derived from the zero-sum game in game theory [62]. $D^{U}$ needs to improve its ability to accurately distinguish between real and generated images. Its loss is composed of two parts:

(8) $$L_{D^{U}}=L_{D_{enc}^{U}}+L_{D_{dec}^{U}}$$

where L represents the loss function, $D_{enc}^{U}$ represents the encoder and $D_{dec}^{U}$ represents the decoder. $D_{enc}^{U}$ plays the same role as the discriminator in the vanilla GAN [4] (see Formula (9)), providing global information based on high-level features.

(9) $$L_{D_{enc}^{U}}=-E_{x\sim P(x)}\left[\log\left(D_{enc}^{U}\left(x\right)\right)\right]-E_{z\sim P(z)}\left[\log\left(1-D_{enc}^{U}\left(G\left(z\right)\right)\right)\right]$$

The decoder part of the discriminator $D_{dec}^{U}$ provides local information based on low-level features through per-pixel analysis of the feature map. Its loss function is as follows:

(10) $$L_{D_{dec}^{U}}=-E_{x\sim P(x)}\left[\sum_{i,j}\log{\left[D_{dec}^{U}\left(x\right)\right]}_{i,j}\right]-E_{z\sim P(z)}\left[\sum_{i,j}\log\left(1-{\left[D_{dec}^{U}\left(G\left(z\right)\right)\right]}_{{}_{i,j}}\right)\right]$$

where *i* and *j* denote the coordinates of the currently processed pixel. 

As gradient normalization is only used as an auxiliary method to improve the training process of $D^{U}$ by controlling the gradient, it does not directly participate in the calculation of the loss. Therefore, it does not change the definition of the loss function.

G learns to generate images that are indistinguishable from real ones by learning the distribution of real samples:

(11) $$L_{G}=-E_{z∼P\left(z\right)}\left[\log D^{U}\left(G\left(z\right)\right)\right]$$

The generator G and discriminator $D^{U}$ enhance each other in the confrontation, and eventually they reach an equilibrium state.

## 4. Experiments

### 4.1. Datasets

To verify the performance of our SUGAN on image generation tasks, we conducted experiments on three datasets in this paper: CIFAR-10 [63], CelebA-HQ [64] and Anime. There are 10 classes in the CIFAR-10 dataset containing 60,000 images with a resolution of 32 × 32 pixels. CelebA is a publicly available dataset commonly used for face generation tasks, provided by the Chinese University of Hong Kong. It contains 202,599 face images with a resolution of 178 × 218 pixels. CelebA-HQ improves on the quality and clarity of CelebA and contains 30,000 high-resolution face images with a resolution of 1024 × 1024 pixels selected from the latter. Anime dataset was crawled from the website Konachan.net, and the images were cropped to the required spatial size, and then manually cleaned. The entire dataset contains 40,000 anime images with a resolution of 256 × 256 pixels.

To standardize the resolutions of high-resolution images input into the model, the images in CelebA-HQ and Anime datasets were scaled to 128 × 128 pixels before the experiments, and the specific information of the three datasets are shown in Table 2. Among them, we use CIFAR-10 for class-conditional image synthesis, and CelebA-HQ and Anime for unconditional image synthesis.

### 4.2. Evaluation Metrics

We use Inception Score (IS) [49] and Fréchet Inception Distance (FID) [50] as the metrics to objectively evaluate the quality and diversity of generated images. For the metric of IS, the higher the evaluation value, the better the experiment result (the symbols of ↑ in the following tables express the same meaning). The situation is the opposite for the metric of FID (the symbols of ↓ in the following tables denote the same meaning). Between the two metrics, FID is considered a more reasonable and comprehensive measure, following the practice in the references of [6,42,50]. It calculates the similarity between real and generated samples by assessing the distance between them in the feature space. In contrast, IS is limited by the recognition capabilities of the Inception classifier: when the generated images do not fall into any category that the classifier can recognize, the IS will be inherently low, even if the generated images are of high quality. Only when the generated images align with the category labels recognizable to the classifier of IS, the IS metric becomes a valuable reference. Therefore, in our experiments, we use FID as the main evaluation metric while using IS as an auxiliary metric. 

### 4.3. Results

#### 4.3.1. Unconditional Image Synthesis

**Figure 6:**
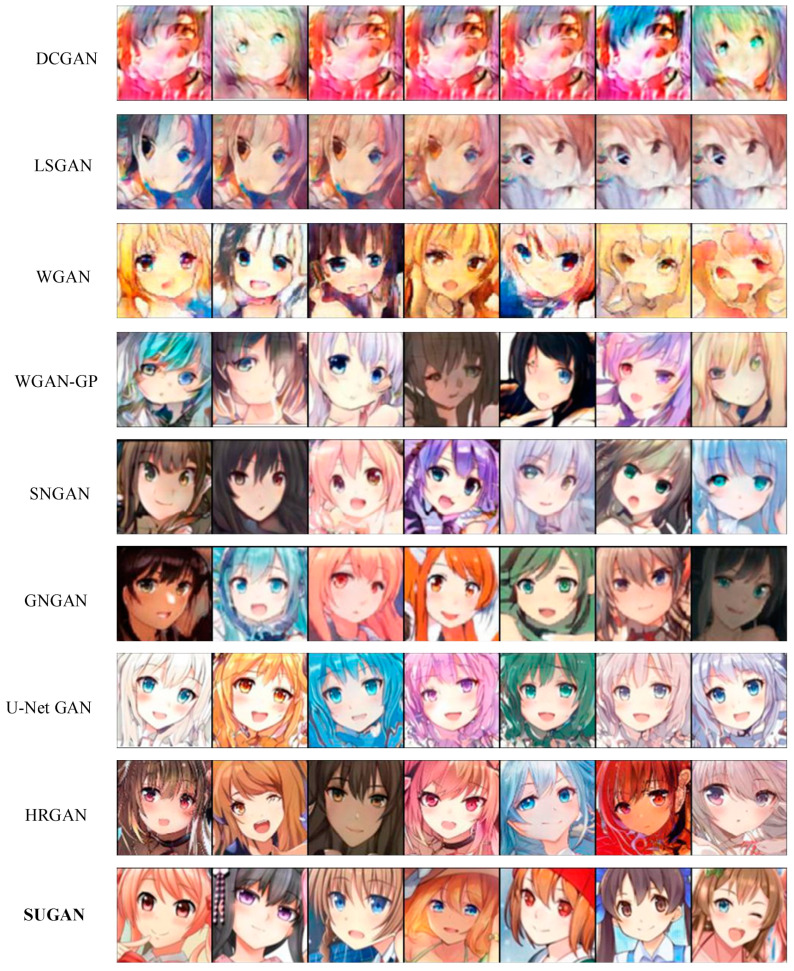
Unconditional image generation results of different models on the Anime dataset.

**Figure 7:**
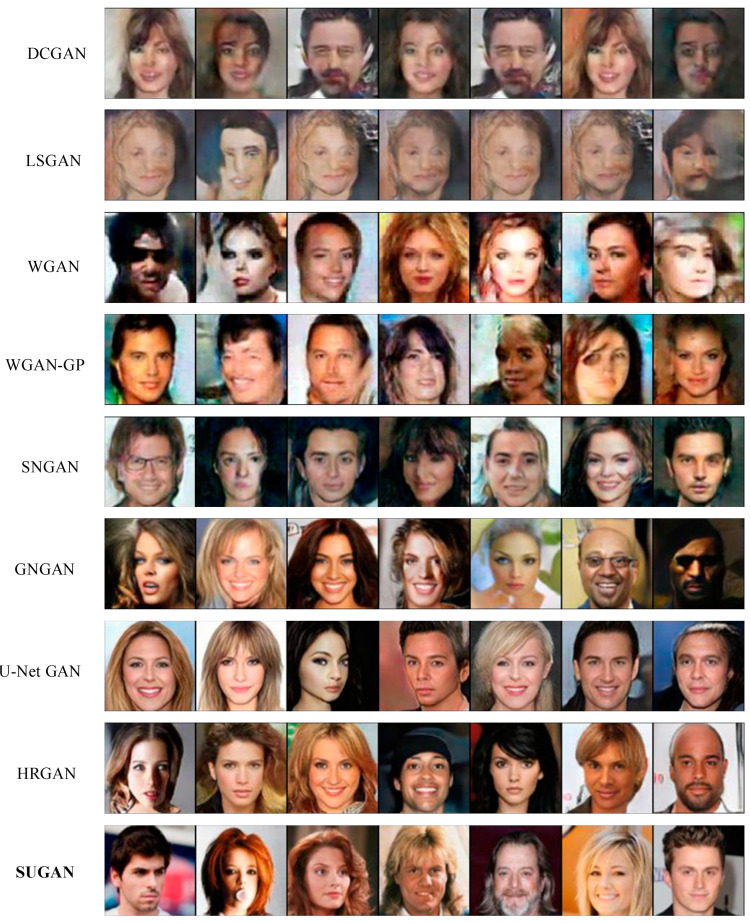
Unconditional image generation results of different models on the CelebA-HQ dataset.

In order to verify the performance of our SUGAN in the unconditional image generation task, we compared it with eight mainstream GANs: DCGAN [35], LSGAN [36], WGAN [54], WGAN-GP [57], SNGAN [47], GNGAN [48], U-Net GAN [46], and HRGAN [65]. All the above models are representative in further exploring the generative potential of GANs. Among them, to improve the performance of GANs, DCGAN, WGAN, and U-Net GAN modify the network architectures, WGAN-GP and SNGAN explore the function of normalization strategies, and LSGAN modifies the loss function. HRGAN presents a novel concept by introducing an image calibration network to enhance the image resolution during the training process of the generator. This approach allows the generator to learn more feature information from higher-resolution images, and thus the quality of the generated images can be greatly improved. The comparison results are shown in Table 3, Figure 6 and Figure 7, where our results are emphasized in bold, which also applies to the remaining figures and tables in this paper. Note that the comparison among U-Net GAN, GNGAN, and our SUGAN constitutes the ablation experiment. 

Table 3 shows the IS and FID values of the above models in unconditional image synthesis, from which it is easy to see that U-Net GAN, HRGAN, and our SUGAN obtain better results than the other methods, and our SUGAN outperform all the compared methods. Some of the generated images are also shown in Figure 6 and Figure 7. Since DCGAN and LSGAN only improve the training stability of GANs to some extent, there are cases of collapse during the training process, leading to blurry generated images (as shown in Figure 6 and Figure 7), low IS values and high FID values. The normalization strategies used in WGAN, WGAN-GP, SNGAN and GNGAN are weight clipping, gradient penalty, spectral normalization, and gradient normalization, respectively. Among the four GAN models, GNGAN generates images with best quality, and IS and FID values are significantly improved (as seen in Table 3), indicating that gradient normalization can bring better performance to GAN models compared with weight clipping, gradient penalty and spectral normalization. However, the images generated by GNGAN are not clear enough, and the overall quality of the images needs to be improved. U-Net GAN has excellent performance in improving the quality of generated images, but the training process of this model is unstable, with serious problems of mode collapse. As shown in Figure 6 and Figure 7, especially in Figure 6, some of the images generated by U-Net GAN show very similar styles, which is a typical manifestation of mode collapse. Besides, as shown in Figure 7, some of the face images generated by U-Net GAN also look unreal, which is caused by unstable training of this GAN model. 

Our SUGAN achieves an FID of 39.40 on the Anime dataset, which is an improvement of 17.89 points over the U-Net GAN. On the CelebA-HQ dataset, our SUGAN also achieves a significant improvement in FID score. By introducing gradient normalization to U-Net GAN, the face images generated by our SUGAN are more excellent in details, textures, and aspects. As shown in Figure 7, the images generated by our SUGAN also looks more realistic. Moreover, the training process of our SUGAN remains stable, which means that the mode collapse problem observed in U-Net GAN is well alleviated.

Although HRGAN proposes a new method to improve the generation capacity of GANs and achieves good results in our experiments, the image calibration network introduced by HRGAN brings more parameters and thus increases the training burden of the GAN model. In contrast, our SUGAN is relatively lightweight and achieves better IS and FID scores (as shown in Table 3) without introducing additional hyperparameters, demonstrating its effectiveness in unconditional image synthesis tasks. It should be noted that the classifier of IS can only identify classes included in the CIFAR-10 dataset. This is the reason why the IS scores in Table 3 (evaluated on the CelebA-HQ and Anime datasets) are much lower than in Table 4 (evaluated on the CIFAR-10 dataset). Therefore, for the unconditional image generation task, we use FID as the main metric (see Section 4.2).

#### 4.3.2. Conditional Image Synthesis

**Figure 8:**
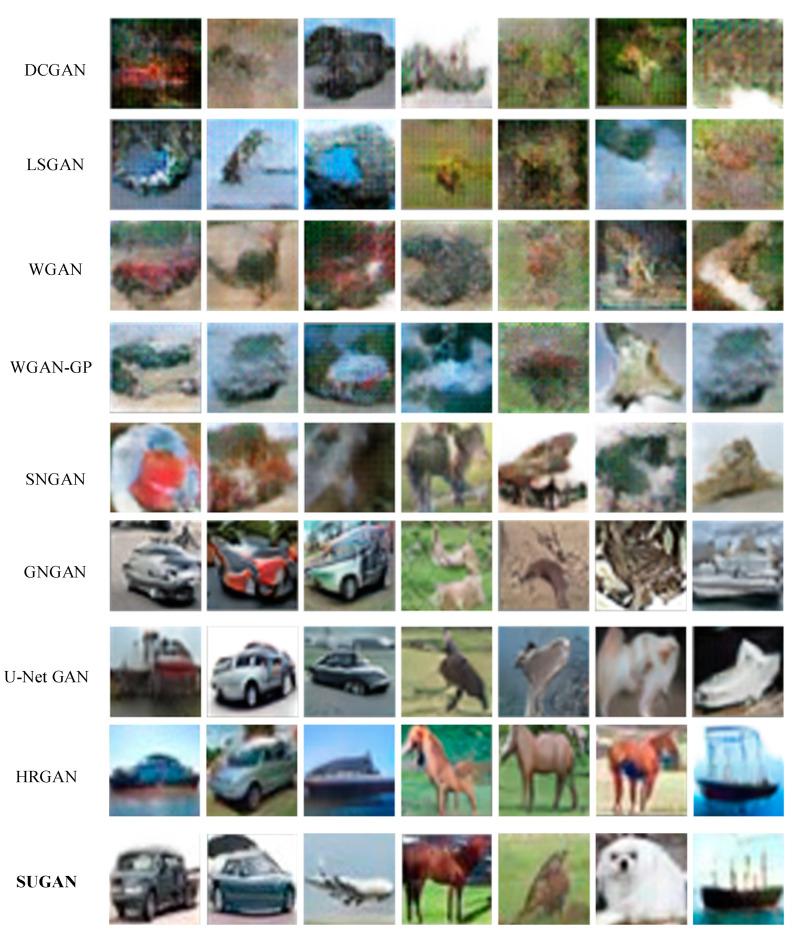
Conditional image synthesis results of different models.

We conducted experiments on the CIFAR-10 dataset to verify the performance of our SUGAN in the task of conditional image synthesis. The comparison results are shown in Table 4, and some of the generated images are shown in Figure 8. Note that because the resolution of the images in the CIFAR-10 dataset is only 32 × 32 pixels, inevitably there is the problem of image blur for the generated images. Therefore, compared with the experiments for the conditional image generation task (see Section 4.3.1), we are more concerned about whether the objects in the generated images look real in this section.

As in the unconditional image synthesis task, in terms of FID scores, our SUGAN again performs best in the conditional image synthesis task (see Table 4). Compared with the U-Net GAN and the second-best model HRGAN, the FID score of our SUGAN is improved by 1.30 and 0.49, respectively. In addition, our SUGAN also outperforms the SNGAN, the GNGAN and the U-Net GAN in terms of the IS score. Our SUGAN does not obtain a clear advantage in IS score compared with the HRGAN: our SUGAN obtains a higher mean (which is desired) but also higher deviation (which is not desired). However, as shown in Figure 8, our SUGAN can generate more realistic images than HRGAN, GNGAN and U-Net GAN, especially when generating images containing animals and vehicles. Therefore, the comparison in this section can also demonstrate the effectiveness of our SUGAN in the conditional image synthesis task.

#### 4.3.3. Comparisons with an Alternative Improved Model

As for alleviating the mode collapse problem, WGAN-GP [57] can also be effective by introducing Wasserstein distance and gradient penalty normalization terms. Therefore, another possible solution is to combine U-Net GAN with WGAN-GP. We implement this improved model and named it UWGAN-GP. 

It should be noted that the combination of spectral normalization and WGAN will result in training failure [48]. This is because the weight clipping in WGAN is sensitive to clipping parameters, and important feature information may be lost due to the forced scaling of weights in the clipping process. Therefore, we implemented UWGAN-GP by introducing the gradient penalty strategy instead of the weight clipping strategy into the discriminator of U-Net GAN, and compared this improved model with our SUGAN.

**Figure 9:**
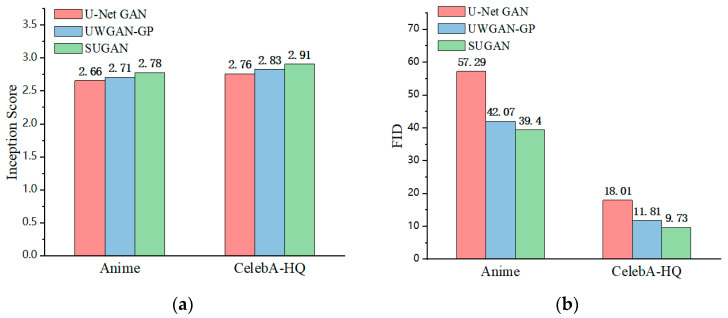
Quantitative comparison of the two improved models on the Anime and CelebA-HQ datasets: (**a**) Comparison results on IS scores, and (**b**) Comparison results on FID scores.

**Figure 10:**
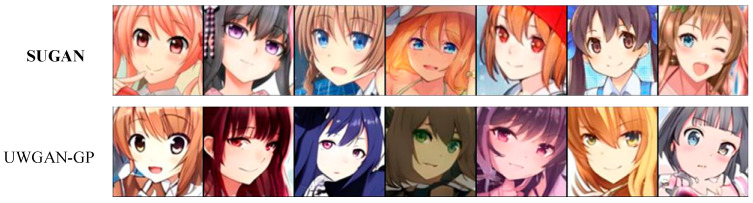
Some generated images of the two improved models on the Anime dataset.

**Figure 11:**
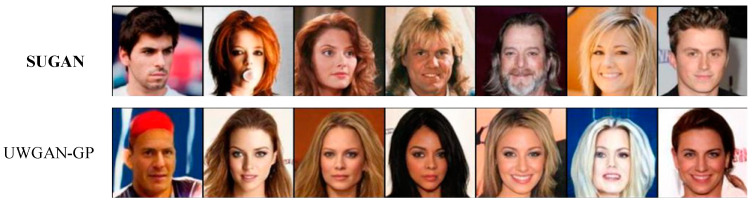
Some generated images of the two improved models on the CelebA-HQ dataset.

**Figure 12:**
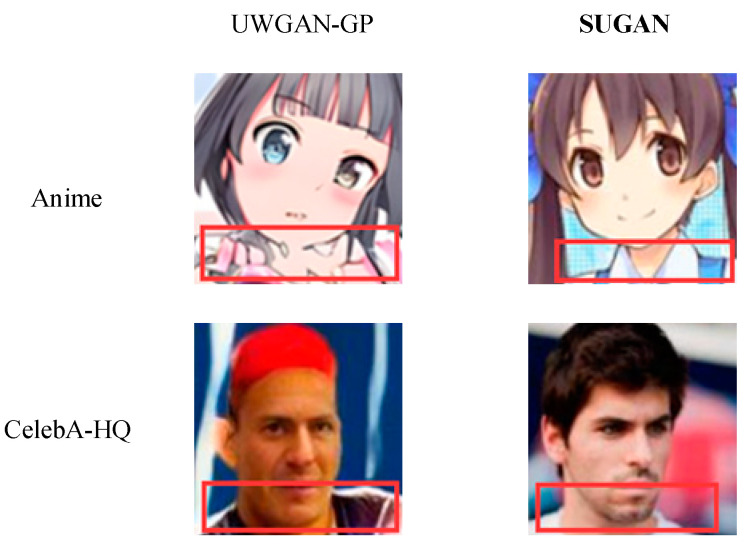
Comparison of the two improved models on restoring clothing details.

To determine whether adding gradient normalization to U-Net GAN or combining U-Net GAN with WGAN-GP is more effective in solving the mode collapse problem, we compared the generated results of our SUGAN and UWGAN-GP on the CelebA-HQ and Anime datasets. The experimental results are shown in Figure 9, Figure 10, Figure 11 and Figure 12. 

As can be seen in Figure 9, on the Anime dataset, UWGAN-GP and our SUGAN improve the IS score by 0.05 and 0.12, respectively, and the FID score by 15.22 and 17.89, respectively, compared to the original U-Net GAN. On the CelebA-HQ dataset, the comparative results are similar. These quantitative comparison results show that both of the two improved models can achieve improvement for image generation quality, and our SUGAN obtains better results.

Figure 10 and Figure 11 show that both of the two improved models also have good performance in alleviating the mode collapse problem. However, the performance of our SUGAN is stronger, which is reflected in their ability of processing image details. Figure 12 presents an enlarged comparison of image details, where we can see that our SUGAN has excellent abilities of processing facial details and clothing details, while UWGAN-GP functions poorly in processing clothing details (see the regions marked by the red rectangles). On the Anime dataset, the clothes of the characters restored by UWGAN-GP are blurry and lack realism. On the CelebA-HQ dataset, the character generated by UWGAN-GP displays white highlights above his cloth, accompanied by blurred edges. Therefore, visual comparison also shows that compared to combining with WGAN-GP, combining with gradient normalization (as our SUGAN) is a better solution to improve the original U-Net GAN.

### 4.4. Hyperparameter Experiments

Batch size has a significant impact on the quality of images generated by the GAN models. Andrew Brock et al. [37] proposed that when training GANs and its derivative models with deep network structures, as the batch size increases, each batch processed contains more image style information, which allows the model to achieve better performance in fewer iterations. However, too large batch size may cause instability during model training and ultimately result in mode collapse.

**Figure 13:**
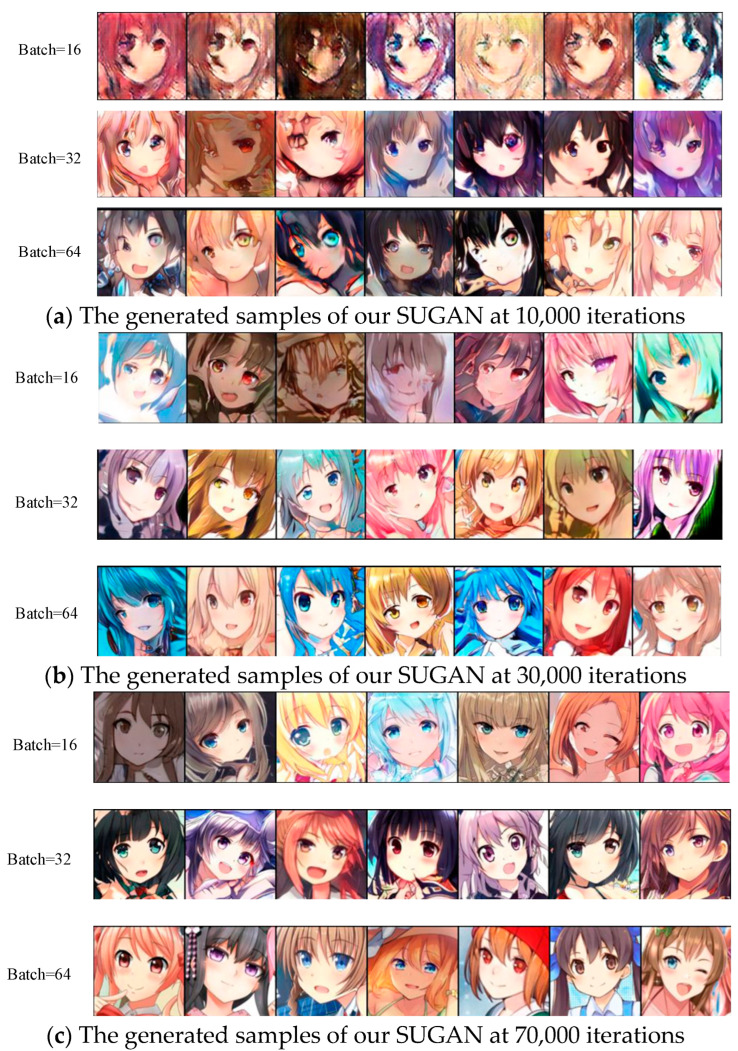
Some of the generated samples of our SUGAN at (**a**) 10,000 iterations, (**b**) 30,000 iterations and (**c**) 70,000 iterations. When the number of iterations is 10,000 and 30,000, the larger the batch size, the higher the quality of the generated images. When the number of iterations is 70,000, the quality of the generated images corresponding to the three batch sizes are similar.

To verify the impact of batch size on the performance of our SUGAN, three different batch sizes were used to train the model on the Anime dataset, and the generated results were recorded when the iteration number was 10,000, 30,000, and 70,000, respectively. Some of the results are shown in Figure 13. In the experiments with 10,000 and 30,000 iterations, the model generates samples of higher quality and diversity when trained with a batch size of 64. When the number of iterations is 70,000, the generated sample quality of the three batch sizes is similar. The above comparison shows that when the iteration number is not very large, the batch size matters a lot, and large batch sizes tend to lead to better results; but as the iteration number becomes larger, the influence of batch size becomes weaker, and relatively small batch sizes can also lead to good results after enough iterations.

From Figure 13, it is also easy to find that our SUGAN performs well for all the three batch sizes, and the training process is stable. The training stability of GAN models comes not only from generators or discriminators, but also from their interactions. A powerful discriminator can make the generator stronger. On the premise of stable training of the generator, the images generated by GANs will be of high quality if the discriminator is very powerful. However, when there is substantial performance gap between the generator and discriminator, the generator cannot cheat the discriminator no matter how it improves itself. And then it will completely fail in the competition against the discriminator, which will result in mode collapse. Although the deep discriminator network with U-Net architecture in our SUGAN is very powerful compared to the generator, with the help of gradient normalization, the training process of our SUGAN is stable, and there is no training collapse problem caused by the generator being completely defeated by the discriminator. This means that even when facing complex datasets, deep network architectures and large batch sizes, our SUGAN can still ensure training stability and image diversity. All the above experimental results (in Section 4, including the current section) also demonstrate the effectiveness of our SUGAN in image generation tasks.

## 5. Conclusions

In order to realize a GAN model with both high image generation quality and stable model training, this paper proposes a stable version of U-Net GAN by applying gradient normalization to its discriminator. The proposed model SUGAN not only improves the generation capability, but also alleviates the mode collapse problem caused by unstable training, i.e., the training process is stable, and the generated samples are of higher quality and more diverse. In all experiments, our SUGAN demonstrates strong generation ability and high training stability.

Although our SUGAN shows good performance if trained with high-resolution datasets, the generated image quality when trained with low-resolution datasets still has a lot of room for improvement. Besides, even though gradient normalization does not introduce additional hyperparameters, compared with other normalization methods, its calculation process is more time-consuming and thus its introduction increases the training burden of the discriminator. Therefore, designing normalization methods with lower computational cost, stronger generative performance and better adaptation to various resolutions is a future research direction for us.

## Figures and Tables

**Table 1:** The comparison of GN, GP, and SN.

Normalization	Model-Wise	Non-Sampling-Based	Hard
GP	√		
SN		√	√
GN	√	√	√

**Table 2:** Details of the datasets used in this paper.

Dataset	Number of Samples	Resolution
CelebA-HQ	30,000	128 × 128
Anime	40,000	128 × 128
CIFAR-10	60,000	32 × 32

**Table 3:** Quantitative results of unconditional image synthesis.

Model	Anime		CelebA-HQ	
	IS ↑	FID ↓	IS ↑	FID ↓
DCGAN	1.40 ± 0.64	223.66 ± 0.37	1.85 ± 0.02	139.96 ± 0.40
LSGAN	1.34 ± 0.09	272.05 ± 0.19	1.74 ± 0.33	192.15 ± 0.19
WGAN	2.21 ± 0.38	120.26 ± 0.81	2.31 ± 0.79	101.34 ± 0.26
WGAN-GP	2.29 ± 0.11	85.61 ± 0.85	2.36 ± 0.12	54.87 ± 0.83
SNGAN	2.37 ± 0.21	73.32 ± 0.81	2.48 ± 0.79	43.23 ± 0.31
GNGAN	2.48 ± 0.66	66.58 ± 0.50	2.52 ± 0.46	24.37 ± 0.79
U-Net GAN	2.66 ± 0.64	57.29 ± 0.81	2.76 ± 0.68	18.01 ± 0.20
HRGAN	2.70 ± 0.05	43.15 ± 0.42	2.81 ± 0.51	12.44 ± 0.16
**SUGAN (ours)**	**2.78** ± **0.13**	**39.40** ± **0.71**	**2.91** ± **0.77**	**9.73** ± **0.94**

**Table 4:** Quantitative results of conditional image synthesis.

Model	CIFAR-10	
	IS ↑	FID ↓
DCGAN	6.46 ± 0.30	38.73 ± 0.61
LSGAN	5.89 ± 0.62	43.08 ± 0.30
WGAN	6.93 ± 0.61	34.60 ± 0.15
WGAN-GP	7.86 ± 0.12	26.01 ± 0.12
SNGAN	8.22 ± 0.05	15.37 ± 0.32
GNGAN	8.49 ± 0.45	11.13 ± 0.83
U-Net GAN	8.55 ± 0.31	10.92 ± 0.75
HRGAN	8.69 ± 0.11	10.11 ± 0.04
**SUGAN (ours)**	**8.75** ± **0.29**	**9.62** ± **0.18**

## Data Availability

The CIFAR-10 dataset used in this paper that is publicly released by Alex Krizhevsky can be downloaded from the link: http://www.cs.toronto.edu/~kriz/cifar.html (accessed on 17 August 2023). The CelebA-HQ dataset used in this paper that is publicly released by The Chinese University of Hong Kong can be downloaded from the link: http://mmlab.ie.cuhk.edu.hk/projects/CelebA.html (accessed on 17 August 2023). The Anime dataset used in this paper can be downloaded from the link: https://aistudio.baidu.com/datasetdetail/110820 (accessed on 17 August 2023). The source code and trained model of our SUGAN are publicly available at https://github.com/ChengsjCV/SUGAN (accessed on 17 August 2023).
